# Application of Tranexamic Acid in Total Knee Arthroplasty – Prospective Randomized Trial

**DOI:** 10.2174/1874325001711011049

**Published:** 2017-08-29

**Authors:** Joao Paulo Fernandes Guerreiro, Bruno Scatolon Badaro, Jose Rodolfo Martines Balbino, Marcus Vinicius Danieli, Alexandre Oliveira Queiroz, Daniele Cristina Cataneo

**Affiliations:** 1 UNIORT.E Orthopedic Hospital, Londrina, Parana state, Brazil; 2 Londrina Evangelic Hospital, Londrina, Parana state, Brazil; 3 Universidade Estadual Paulista Julio de Mesquita Filho, Botucatu, Sao Paulo state, Brazil

**Keywords:** Arthroplasty, Arthritic knee, Bleeding, Tranexamic acid, Pain, Haemoglobin

## Abstract

**Background:**

The use of tranexamic acid (TXA) in total knee arthroplasty (TKA) has shown good results. Bleeding may cause local complications consequently greater pain and reduced function postoperatively. No study has related the use of TXA to these facts.

**Objective:**

The aim was to evaluate the effects of TXA haemoglobin, Western Ontario and McMaster Universities Index (WOMAC), pain intensity and flexion gain after TKA.

**Methods:**

43 patients were randomized and then underwent TKA. TXA was applied to 22 of these patients before closure of the joint capsule. Haemoglobin measurements (mg/dL) were taken preoperatively and 24 and 48 hours after surgery. The WOMAC questionnaire and pain visual analogue scale (VAS) were applied, and flexion gain was measured up to the second postoperative month. Statistical analysis compared the results to determine whether there were differences between the groups for each of the evaluated times.

**Results:**

There were differences in favour of the drug 48 hours postoperatively for the haemoglobin variable (p = 0.01), in pain evaluation, 24 and 48 hours, postoperatively (p < 0.01) and in flexion gain, 24 hours after surgery (p = 0.03). There were no significant differences between the groups in the haemoglobin evaluation 24 hours postoperatively, in pain assessment 7 days, 21 days and 2 months, postoperatively, in flexion gain 48 hours, 7 days, 21 days and 2 months, postoperatively and in WOMAC after 2 months.

**Conclusion:**

In addition to reducing bleeding, topical TXA improved pain and increased flexion gain in the first hours after TKA.

**Trial Registration:**

RBR-9b4qgq

## 1 INTRODUCTION

Total knee arthroplasty (TKA) is the ideal surgical option for treating severe pain in the arthritic knee [1]. According to the literature, approximately 20% of patients with surgical indications have preoperative anaemia and, therefore, are at a relatively higher risk of requiring blood transfusion [2, 3].

**Fig. (1):**
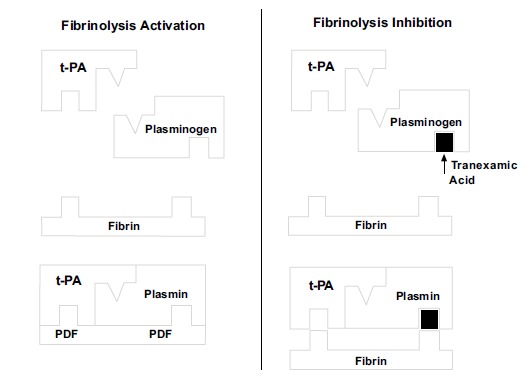
**Action mechanism of tranexamic acid (TXA).** The site in plasminogen where fibrin binds is occupied by TXA, preventing fibrinolysis. T-PA - Tissue plasminogen activator; FDP - fibrin degradation products. Source: Santos *et al.* (19).

Intraoperative homeostatic control is important and can avoid possible procedural and transfusion complications in patients with anaemia in the postoperative period [2, 4]. It is known that blood component administration increases the risk of infections and immune reactions [4, 5]. Several haemostatic control strategies have proven effective in recent years [6, 7]. Classical strategies for the control of intraoperative bleeding include the use of a pneumatic tourniquet, anaesthesia and low-pressure haemostasis [4, 8]. Recently, tranexamic acid (TXA) has been used as an adjuvant to such measures [6-18]. TXA is an analogue of lysine and inhibits fibrinolysis and blocks, with high affinity, the lysine to plasminogen binding sites, thereby preventing formation of the complex between plasminogen, fibrin and tissue plasminogen activator [19] (Fig. **1**). TXA is a synthetic product that is inexpensive and easily accessible [8, 10-12], with good results in cardiac surgery. It has attenuated haemostatic disorders for over 20 years [20].

Orthopaedics initially hesitated to use this substance, as there was a lack of information regarding the safety of TXA use in relation to thromboembolic events [7, 11, 21, 22]. However, more recent studies have demonstrated the benefits of TXA in TKA, revealing different administration routes and therapeutic dosages [5-7, 9, 10, 17, 23].

Intra-articular administration of TXA in TKA has shown good results, effectively reducing the decrease in haemoglobin, the blood loss through drainage and the need for transfusion for up 48 hours, postoperatively [14, 16]. It is known that persistent bleeding during the postoperative period may cause local complications, such as haematoma, seroma and consequently greater pain and reduced function postoperatively [24, 25]. No study has related use of TXA to these facts.

Therefore, the hypothesis of this study was that, in addition to reducing bleeding, the topical application of TXA at the end of surgery before closure of the joint capsule and surgical incision would be effective in controlling pain and improving functional recovery and flexion gain in the postoperative period of TKA.

## 2 OBJECTIVE

The objective was to evaluate the effects of TXA on serum haemoglobin, the Western Ontario and McMaster Universities Index (WOMAC), pain intensity and flexion gain after TKA.

## 3 METHODS

The study was conducted in the city of Londrina-PR, at the Brotherhood of Santa Casa de Londrina, Philanthropic Hospital (Irmandade da Santa Casa de Londrina, Hospital Filantrópico), a reference in high-complexity orthopaedics and traumatology services within the Unified Health System (Sistema Único de Saúde - SUS). This study was undertaken after approval by the Research Ethics Committee of the institution, which is linked to the National Research Ethics Commission (Comissão Nacional de Ética em Pesquisa - CONEP). This study was approved under protocol number 634815 on 05/05/2014. Trial Registration: RBR-9b4qgq.

A total of 43 patients with indications for TKA, treated at the outpatient clinic of the institution, were selected between June 2014 and October 2015. The selected patients were duly informed about the project and signed terms of free and informed consent. Inclusion criteria were patients of either gender with the presence of tricompartmental knee osteoarthritis with varus deformity, with indications for TKA. Exclusion criteria were patients with major deformities that would lead to bone cuts or release of a more extensive area of soft tissue; presence of inflammatory diseases; patients who had undergone previous surgeries of the same knee; use of anticoagulation medication up to seven days before surgery; and patients with history of atrial fibrillation, deep vein thrombosis or prior pulmonary embolism.

**Fig. (2):**
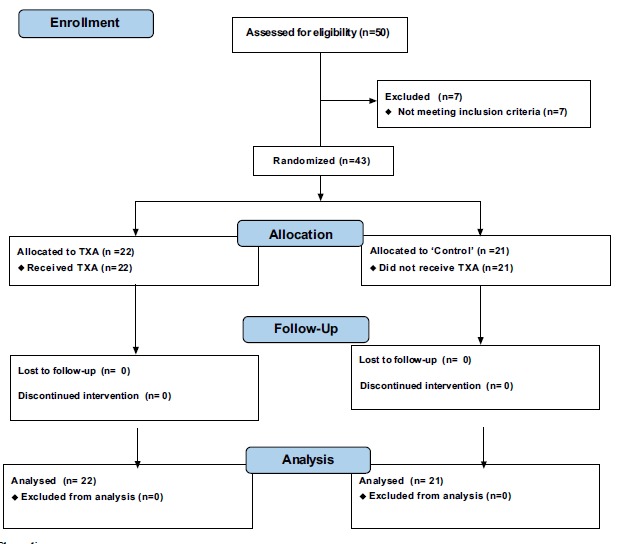
Flow diagram.

The subjects were divided into 2 groups (Fig. **2**): those using TXA and a control group (no applied substance). Allocation was randomized by the surgeon, and the subjects were monitored for 2 months. The groups were named “Control” (21 patients undergoing total knee prosthesis without application of TXA or any other intra-articular sealant substance) and TXA (22 patients undergoing total knee prosthesis and intra-articular application of TXA during the TKA procedure). The dose chosen for topical treatment followed the existing literature data on the use of TXA in total knee prosthesis and was 1.0 g (4 x 5 ml ampoules with a concentration of 50 mg/ml) [26, 27]. Patients were not told to which group they belonged and were completely unaware of this information until the end of the project.

The following data on the pre- and postoperative periods were recorded (Table **1**):

1. Haemoglobin (Hb) levels preoperatively and 24 and 48 hours after surgery.
2. Reports of clinical flexion gain examination using a goniometer for evaluations 24 hours, 48 hours, 7 days, 21 days and 2 months after surgery.
3. Pain evaluation using a visual analogue scale (VAS), which consists of a 1-dimensional instrument containing an 11-point scale (0-10), where zero is rated as no pain and 10 the worst pain one has ever felt. Patients selected a single number that best represented the intensity of their pain at the following postoperative time intervals: 24 and 48 hours, 7 days, 21 days and 2 months after surgery.
4. Evaluations of knee function, preoperatively and 2 months after surgery, using the “WOMAC” instrument, were translated and validated for the Portuguese language [28].

**Fig. (3):**
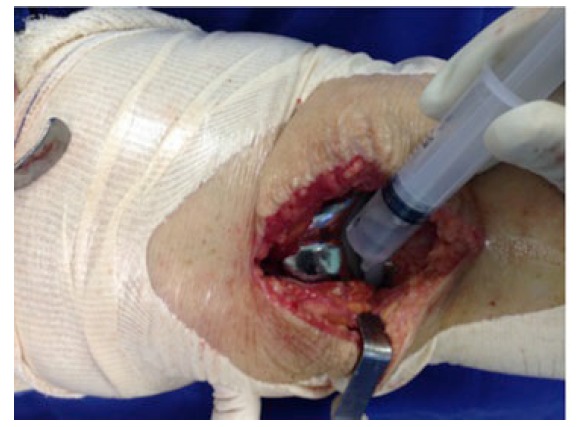
Application of tranexamic acid before capsule closure.

**Fig. (4):**
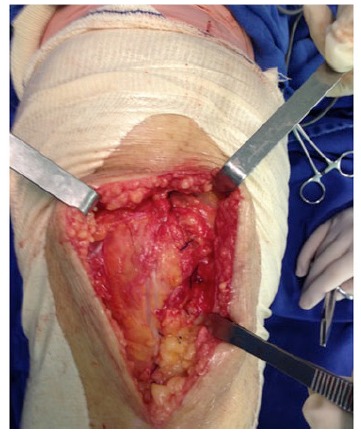
Closing of the joint capsule.

The surgical technique was used documented within the current literature, with subarachnoid anesthesia, patellar medial access and application of TXA all over the exposed joint in the selected cases using a syringe (Fig. **3**) and maintained for 5 minutes. After this period, the joint capsule and incision were closed without drain (Fig. **4**). All procedures were performed by the same surgeon and with the same instrument and implant (Meta Bio Ltd., Rio Claro, São Paulo, Brazil).

Postoperatively, during hospitalization, the analgesics used included 1 g of intravenous dipyrone every 6 hours and 50 mg of tramadol hydrochloride every 8 hours. Patients with pain scores above 7 on the numerical pain scale received 4 mg of intravenous morphine every 4 hours. After discharge, 1 g of dipyrone was prescribed orally every 6 hours in case of pain, and 50 mg of tramadol hydrochloride was administered orally every 6 hours if the pain remained with the use of dipyrone. All patients received as prophylaxis for deep venous thrombosis a dose of 40 mg of enoxaparin 12, 24 and 48 hours after surgery and were prescribed 10 mg Rivaroxaban daily for 10 days at home. Antibiotic prophylaxis was achieved with 2 g intravenous cefazolin during anaesthetic induction and 1 g of cefazolin every 8 hours for 48 hours. The dressing was changed in the hospital on the 2^nd^ postoperative day, every day at home and at the clinic on the 7^th^ day. The stitches were removed on the 21^st^ postoperative day. Patients used a walker for 21 days with a full load from the 2^nd^ postoperative day. Physical therapy was also initiated on admission and was maintained until the second month, postoperatively, 3 times a week with a physical therapy protocol focusing on analgesia, gain in flexion and walking training. Radiological knee examinations were performed in the immediate post-operative period and upon return to the clinic 2 months later.

## 4 STATISTICAL ANALYSIS

Calculation of the statistical power of the sample was achieved using the *sampsi* command of the STATA software package, version 11, based on the method proposed by Frison and Pocock [29] for a group comparison design with repeated measures. 5% significance level was adopted, and upon varying the test power, we found that 20 patients per group would guarantee a power of at least 95% for comparisons [29].

Comparisons between the 2 groups and times with respect to all variables were performed using mixed effect linear regression models (random and fixed effects) [30]. The orthogonal contrast post-test was used for comparisons. Intergroup comparisons regarding changes in Hb and WOMAC times were performed using Student’s t test. The significance level adopted for all comparisons was 5%.

## 5 RESULTS

The mean age of the participants was 68.74 (55-86) years. The mean ages were 68.36 (55-81) years in the TXA group and 69.14 (55-81) years in the control group. The average operating time was 92.9 (80-105) minutes; 94.3 (80-105) minutes in the TXA group and 91.5 (85-95) minutes in the control group. There were 11 male patients; 4 in the TXA group and 7 in the control group Table (**1**).

None of the study participants required blood transfusion. The criterion used to determine transfusion was a haemoglobin value of less than 7 mg/dl in symptomatic patients during the postoperative period. One patient in the TXA group presented dehiscence in the surgical wound and superficial infection, which was treated with dressings and oral antibiotics until their total healing. There were no cases of thromboembolism.

Tables (**2** and **3**) shows that there were statistically significant differences in favour of the group using TXA 48 hours postoperatively for the haemoglobin variable (p = 0.01), 24 and 48 hours postoperatively for pain evaluation (p <0.01) and 24 hours postoperatively for flexion gain (p = 0.03). There were no significant differences between the groups in the haemoglobin evaluation 24 hours postoperatively, in pain assessment 7 days, 21 days and 2 months postoperatively, in flexion gain 48 hours, 7 days, 21 days and 2 months postoperatively and in the WOMAC questionnaire 2 months postoperatively.

## 6 DISCUSSION

The topical administration of 1 g of TXA was effective in reducing bleeding based on the haemoglobin level. This fact, demonstrated again in this study, was already known [16, 26]. In published studies, the most common administration route for the application of TXA in TKA has been intravenous. Intravenous administration in a single dose or repeated doses was used initially in orthopaedic procedures, based on previous studies in cardiac surgery [17]. However, there is evidence demonstrating that only a fraction of the injected drug reaches the target tissue, which in turn reduces its effectiveness [10]; that up to 95% of the drug can be eliminated in the urine; and that in patients with impaired kidney function, the dosage needs to be corrected [19]. When administered in high doses or injected quickly, minor gastrointestinal symptoms, such as nausea and vomiting, have been reported [31, 32]. Intraarticular use generates cost savings because lower doses can be used, without systemic side effects, and also because the surgeon can apply it him- or herself.

Pain, as measured on the visual analogue scale, showed a statistically significant difference (p < 0.01) from 24 to 48 hours postoperatively in the group using TXA. The flexion range of motion analysis showed a statistically significant difference in the group that used TXA (p = 0.03). Pain control and range of motion gain are important goals after the knee arthroplasty procedure. Immediate postoperative pain impairs mobility and reduces the patient's ability to cooperate with rehabilitation treatment [33]. When experiencing postoperative pain, the patient's difficulty in activities of daily living, physical dependence and motion restriction of the operated limb increase their anxiety and discouragement, which can be a complicating factor in the rehabilitation process and may also increase the patient's perception of pain [34]. Alternatively, the use of opioid analgesics for pain control after surgery is recommended; however, a systematic review determined that they are associated with several undesirable side effects, including nausea, vomiting, hypotension, urinary retention, delirium and increased infection rates, which consequently generate an increase in the length of hospital stay and an additional financial cost to the procedure [18]. The use of a less aggressive surgical approach, for example, is now widespread for this purpose but may lead to increased bleeding [35]. In a previous study, our group showed that the application of platelet-rich plasma in total knee replacement surgery reduced postoperative pain but did not reduce bleeding [36]. The literature does not show that TXA is effective in controlling pain or movement gain, as our study has shown, in the first 48 hours after the procedure.

The result obtained in the first 48 hours after the procedure, with advantages in relation to pain and gain in range of motion, was not maintained in the outpatient evaluations in the first months. Knee function, analysed using the WOMAC questionnaire, did not show a better gain in that period than that of the control group.

We believe that the surgeon's and the researcher's knowledge regarding which subjects were cases and which were controls during the procedure and the short follow-up time may be considered weaknesses of the study. A multicentre study with a greater number of individuals may provide better evidence on the subject.

## CONCLUSION

In the manner in which it was used, in addition to reducing bleeding, TXA improved pain and increased flexion gain in the first hours after TKA. There were no differences between groups in terms of pain evaluation, flexion gain and knee function beyond the first 48 hours after the procedure.

## Figures and Tables

**Table 1:** Spreadsheet Model used for data collection of the Parameters analysed at the different time intervals (pre- and post-operative).

	Pre-operative	24 h post-operative	48 h post-operative	7 days post-operative	21 days post-operative	2 months post-operative
Hb	X	X	X			
Flexion	X	X	X	X	X	X
Pain	X	X	X	X	X	X
WOMAC	X					X

Hb: Haemoglobin, Flexion: Flexion gain, Pain: Pain based on a visual analogue scale, WOMAC: Western Ontario and McMaster Universities Index.

**Table 2:** Mean, maximum and minimum values for age, gender distribution and operating time among patients.

	TXA Group	Control Group	Total
Number of patients	**22**	**21**	**43**
Age (years)	**68.36 (55-86)**	**69.14 (55-81)**	**68.74 (55-86)**
Gender (M/F)	**4/18**	**7/14**	**11/32**
Operating time (minutes)	**94.3 (80-105)**	**91.5 (85-95)**	**92.9 (80-105)**

**Table 3:** Comparison between the TXA and control groups.

	TXA Group	Control Group	P Value
Fall in Hb 24 h	**0.98 (0.10-2.10)**	**1.38 (0.20-3.50)**	**p>0.05 (0.10)**
Fall in Hb 48 h	**1.53 (0.40-3.30)**	**2.28 (0.60-4.00)**	**p<0.05** **(0.01)**
Mean pain 24 h	**5.23 (1-9)**	**6.33 (4-8)**	**p<0.05** **(<0.01)**
Mean pain 48 h	**2.45 (0-4)**	**3.81 (2-6)**	**p<0.05** **(<0.01)**
Mean pain 7 days	**1.82 (0-3)**	**2.38 (1-3)**	**p>0.05 (0.08)**
Mean pain 21 days	**1.32 (0-3)**	**1.67 (0-3)**	**p>0.05 (0.26)**
Mean pain 2 months	**1.05 (0-4)**	**1.33 (0-3)**	**p>0.05 (0.35)**
Flexion gain 24 h	**71.82 (50-90)**	**63.81 (40-90)**	**p<0.05** **(0.03)**
Flexion gain 48 h	**80.45 (60-100)**	**80.00 (60-90)**	**p>0.05** **(0.88)**
Flexion gain 7 days	**87.95 (60-100)**	**86.43 (80-100)**	**p>0.05 (0.65)**
Flexion gain 21 days	**94.77 (80-110)**	**95.48 (80-110)**	**p>0.05** **(0.86)**
Flexion gain 2 months	**100.23 (80-120)**	**97.38 (80-115)**	**p>0.05** **(0.42)**
WOMAC 2 months	**45.77 (21-60)**	**45.10 (33-54)**	**p>0.05** **(0.19)**

TXA: Tranexamic acid, Hb: Haemoglobin, WOMAC: Western Ontario and McMaster Universities Index.
